# Threshold-Free Measures for Assessing the Performance of Medical Screening Tests

**DOI:** 10.3389/fpubh.2015.00057

**Published:** 2015-04-20

**Authors:** Yan Yuan, Wanhua Su, Mu Zhu

**Affiliations:** ^1^School of Public Health, University of Alberta, Edmonton, AB, Canada; ^2^Department of Mathematics and Statistics, MacEwan University, Edmonton, AB, Canada; ^3^Department of Statistics and Actuarial Science, University of Waterloo, Waterloo, ON, Canada

**Keywords:** low prevalence rate, area under the ROC curve, average positive predictive value, biomarker, mammography

## Abstract

**Background:**

The area under the receiver operating characteristic curve (AUC) is frequently used as a performance measure for medical tests. It is a threshold-free measure that is independent of the disease prevalence rate. We evaluate the utility of the AUC against an alternate measure called the average positive predictive value (AP), in the setting of many medical screening programs where the disease has a low prevalence rate.

**Methods:**

We define the two measures using a common notation system and show that both measures can be expressed as a weighted average of the density function of the diseased subjects. The weights for the AP include prevalence in some form, but those for the AUC do not. These measures are compared using two screening test examples under rare and common disease prevalence rates.

**Results:**

The AP measures the predictive power of a test, which varies when the prevalence rate changes, unlike the AUC, which is prevalence independent. The relationship between the AP and the prevalence rate depends on the underlying screening/diagnostic test. Therefore, the AP provides relevant information to clinical researchers and regulators about how a test is likely to perform in a screening population.

**Conclusion:**

The AP is an attractive alternative to the AUC for the evaluation and comparison of medical screening tests. It could improve the effectiveness of screening programs during the planning stage.

## Introduction

Screening is an important clinical tool for secondary prevention, which aims at detecting latent conditions or diseases at an early asymptomatic stage. The goal of screening is to facilitate intervention and to improve outcomes (1). The clinical validity of a screening test refers to its ability to *detect* or *predict* the clinical disorder of interest (2). That is, for clinicians, the utility of a screening test is determined by its ability to predict the disorder, i.e., the probability that a subject has the disorder given the screening test result (positive predictive value, PPV). Clinicians recognize that the PPV of a screening test is an important metric partially because of the typical *low prevalence* of the disease in a screening population (3). However, the current performance metrics that evaluate and compare screening tests at the pre-clinical stage do not reflect the prevalence of a disease.

Some screening tests are simply diagnostic tests used on the asymptomatic population. For example, mammography is used at the population-level as a screening test for breast cancers as well as the first diagnostic imaging test for symptomatic patients. Thus, it is not surprising that the same metrics for evaluating *diagnostic* tests have been adopted for evaluating *screening* tests. Commonly used metrics for screening tests include sensitivity, specificity, positive and negative predictive values, positive and negative diagnostic likelihood ratios, among others (4). All the aforementioned metrics require the underlying test to make a binary decision, that is, whether the subject is or is not test-positive. Thus, a decision threshold is needed when the underlying test provides continuous or ordinal measurements. Since different thresholds result in changing values of these metrics, the receiver operating characteristic (ROC) curve that traces the tradeoff between sensitivity and specificity as decision thresholds vary is currently the most popular tool to describe the performance of such tests (4). The area under the ROC curve (AUC) is arguably the most widely used threshold-free numeric index of the ROC curve. It summarizes the performance of a *diagnostic* test over its full range of values instead of at a single threshold. To be considered better or adequate and to be implemented in clinical practice, new tests at the research stage are often expected to show larger or equivalent AUC values when compared to the standard test.

Given that the administration of screening tests is in *asymptomatic* populations where many screened diseases have low prevalence, we sought to evaluate the adequacy of the AUC in this setting against an alternate metric, a weighted average of positive predictive values (AP). Like the AUC, the AP is a single numeric performance metric that does not require a decision threshold; thus, it evaluates the test over its full range of values. Like the AUC, the AP is the area under the precision-recall (PR) curve, which plots precision (same as the PPV) versus recall (same as sensitivity). PR curves are widely used in information retrieval and have been used as an alternative to ROC curves for applications to heavily unbalanced data (5, 6). It has been shown that two retrieval algorithms comparable in the ROC space can be very different in the PR space when there are many more observations from one class than the other (7, 8). Screening tests operating under low prevalence rates are similar to retrieval algorithms applied to heavily unbalanced data. Mathematically, the following two questions are equivalent:

1. How effective can a screening test tell if a patient is diseased or not?
2. How effective can a retrieval algorithm tell if a document is relevant or not?

The AUC lacks sensitivity in identifying cases (9). Wald and Bestwick argued that the AUC is an unreliable performance measure for screening tests (10). By varying the SD and the mean for the test scores of diseased individuals, they were able to construct tests having the same AUC but vastly different detection rates at given false positive levels. Later in this article, we will show that the AP behaves much better in that situation.

Our objective is to explore (1) the relationship between the two threshold-free evaluation metrics, the AUC and the AP, and (2) the possibility of using the AP for improving decision making regarding screening tests. In the following sections, we first contrast the AP and the AUC for evaluating screening tests using an illustrative example with hypothetically different prevalence rates. Then, we define various quantities of interest using a common set of notations, in order to gain insight into the connection between the AP and the AUC. We derive an asymptotic variance formula for the AP in the next section, and demonstrate its usage with a screening mammography example. Finally, we summarize our findings and discuss why the AP has advantages over the AUC when evaluating screening tests as opposed to diagnostic tests.

## Illustrative Example

Before we give a formal mathematical definition for the AP in the Section “Definitions”, let us look at the following illustrative example that compares the AP with the AUC for identifying possible biomarkers for screening.

By analyzing serum samples obtained from the Virginia Prostate Center Tissue and Body Fluid Bank, Adam et al. (11) identified 779 potential protein biomarkers using a technology called “surface-enhanced laser desorption/ionization time-of-flight mass spectrometry” (12). Wang and Chang (13) used this data set to illustrate the partial AUC. We focused on the late-stage prostate cancer patients (*n*_1_ = 83) and the normal individuals (*n*_0_ = 82) in the data set, although the original data set also included patients with early-stage cancer and patients with benign prostate hyperplasia.

**Figure 1:**
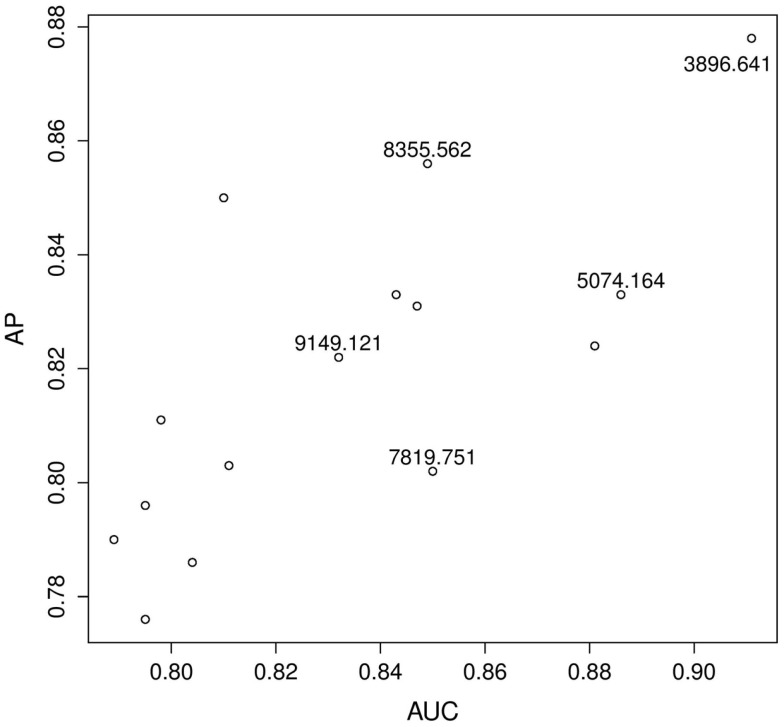
**Prostate cancer example**. Top 15 biomarkers according to the AP. Biomarkers are not labeled unless they are explicitly mentioned in the text.

Figure 1 shows the estimated AP versus the estimated AUC for the top 15 biomarkers as ranked by the estimated AP. Some biomarkers are ranked similarly on both scales, e.g., according to both the AP and the AUC, 3896.641 is a top biomarker. Other biomarkers are ranked very differently. For example, according to the AUC, there is little performance difference between 8355.562 and 7819.751 whereas, according to the AP, 8355.562 has better predictive power. Therefore, it is clear that these two metrics are measuring different aspects of a test. Our question is: what are the implications of these differences in the screening setting?

**Figure 2:**
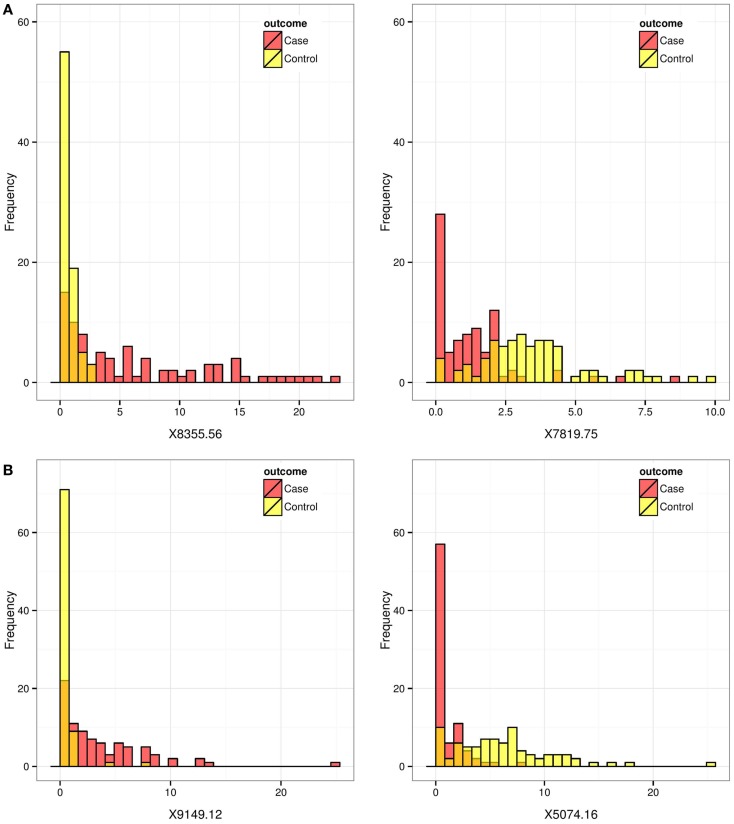
**Prostate cancer example**. Histograms for biomarkers that are ranked differently by the AP and by the AUC. Red and yellow histograms represent cases and controls, respectively. Pair **(A)** (8355.562, 7819.751) scored similarly on the AUC-scale but very differently on the AP-scale. Pair **(B)** (9149.121, 5074.164) scored somewhat similarly on the AP-scale but very differently on the AUC-scale.

**Figure 3:**
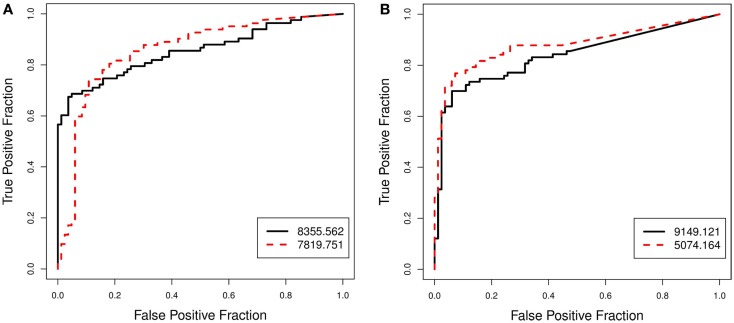
**Prostate cancer example**. Comparison of ROC curves for biomarkers that are ranked differently by the AP and by the AUC. Pair **(A)** (8355.562, 7819.751), which scored similarly on the AUC-scale but very differently on the AP-scale, is shown in **(A)**. Pair **(B)** (9149.121, 5074.164), which scored somewhat similarly on the AP-scale but very differently on the AUC-scale, is shown in **(B)**.

To explore and investigate the implications, we selected two pairs of biomarkers: pair A (8355.562 and 7819.751), which had very similar AUC scores but very different AP scores; and pair B (9149.121 and 5074.164), which scored similarly on the AP-scale but very differently on the AUC-scale. Figure 2 displays the histograms of the raw data for the two selected pairs. Figure 3 compares the resulting ROC curves of the two pairs. We can see clearly from Figure 3 that the two biomarkers in pair A have qualitatively different ROC curves, yet their AUC values are very similar. For the two biomarkers in pair B, one can immediately discern that 5074.164 has a larger area under its ROC curve (i.e., larger AUC), yet their AP-values are similar.

In this example, biomarkers were evaluated under a case–control design (*n*_1_ = 83 ≈ 82 = *n*_0_), which is typical in clinical research settings for evaluating biomarkers and tests. Since the purpose of this case–control study is to identify biomarkers as future screening tools, the prevalence of the disease is expected to be much lower. To see how the relative evaluations may change as measured by the AP and the AUC for the biomarker pairs A and B, we conducted a simple thought experiment by duplicating the control subjects to lower the prevalence (Table 1).

**Table 1:** **Prostate cancer example**.

Biomarkers		AUC			AP		
		*n*_0_ × 1 (π ≈ 0.5)	*n*_0_ × 10 (π ≈ 0.09)	*n*_0_ × 100 (π ≈ 0.01)	*n*_0_ × 1 (π ≈ 0.5)	*n*_0_ × 10 (π ≈ 0.09)	*n*_0_ × 100 (π ≈ 0.01)
A	8355.562	0.849	0.783	0.783	0.856	0.606	0.571
	7819.751	0.850	0.857	0.857	0.802	0.370	0.062
B	5074.164	0.886	0.869	0.869	0.833	0.306	0.043
	9149.121	0.832	0.793	0.793	0.822	0.512	0.225

*A simple thought experiment showing changes in the estimated AUC and AP as a result of artificially inflating the number of control subjects (*n*_0_) to mimic real-life screening settings, where the prevalence (π) of disease is low*.

The AUC should not change when the disease prevalence changes because it is independent of prevalence. The differences observed in the estimated AUCs (Table 1) when the prevalence is lowered from about 0.5 to 0.09 are due to the way the tied scores were handled in estimating the AUC, a minor detail, which we will not discuss here. We can see that, for pair A (8355.562, 7819.751), while the marker 8355.562 scores slightly higher on the AP-scale when the prevalence is 0.5, the difference (Δ) between the two markers becomes much more dramatic on the AP-scale when the prevalence is reduced from 0.5 (Δ = 0.05) to 0.09 (Δ = 0.23) and then to 0.01 (Δ = 0.5). For pair B (9149.121, 5074.164), even though the marker 5074.164 has a higher AUC, the estimated AP is more or less indifferent between the two markers when prevalence is at 0.5. But when the prevalence is reduced, the AP actually starts to favor the marker 9149.121. Our experiment shows that, according to the AP metric, when the prevalence is low and the goal is to identify diseased subjects, biomarkers 8355.562 and 9149.121 perform much better than biomarkers 7819.751 and 5074.164, respectively. Among the four biomarkers, the estimated AP of biomarker 8355.562 decreases least drastically as the prevalence rate decreases from 0.5 to 0.01, showing that its predictive power is best preserved for identifying diseased subjects as the prevalence decreases.

## Definitions

In this section, we define various concepts associated with evaluating the effectiveness of a screening test. Our objective is to formally define the AUC and the AP so that they can be studied together. In order to do so, it is convenient to start with the so-called hit function.

### Population version and continuous scores

Suppose there are a total of *N* subjects in a target population, *N_1_* of which have the disease of interest and the rest *N_0_ =* *N* − *N_1_* of which do not have the disease. For every subject, a screening test produces a score, *x*, with which we can rank (or order) the subjects – e.g., the higher the score (larger *x_i_*), the more likely the subject is to have the disease, and vice versa.

Let *i* denote the *ordered* subject index, that is, *x*_1_ ≥ *x*_2_ ≥ … ≥ *x*_N_. If the threshold is set at x*_k_*, then all subjects with scores greater than or equal to x*_k_* are classified as diseased by the test and all those with scores less than x*_k_* are classified to be non-diseased.

Let

- π be the prevalence of the disease in the target population – mathematically, π ≡ *N*_1_/*N* = *P*(*Y*  = 1) where *Y* indicates the disease status, 1 for diseased and 0 for non-diseased;
- *d*(*k*) be the number of subjects with scores greater than or equal to *x*_k_ – as *x*_k_ takes on decreasing values from slightly above *x*_1_ to *x*_N_, *d*(*k*) increases from 0 to *N*;
- *m*(*k*) be the number of truly diseased subjects in those *d*(*k*) subjects – as x*_k_* takes on decreasing values from slightly above *x*_1_ to *x*_N_, *m*(*k*) increases from 0 to *N*_1_;
- *s* be the probability that a subject has a test score greater than or equal to x*_k_* – mathematically,
  $$s\equiv d\left(k\right)∕N=P\left(X\geq x_{k}\right),$$

and as *x*_k_ takes on decreasing values from slightly above *x*_1_ to *x*_N_, *s* increases from 0 to 1.

Then, the hit function is

$$h\left(s\right)\equiv m\left(k\right)∕N,$$

i.e., the probability that a subject with a test score greater than or equal to *x*_k_ is diseased.

When *N* is relatively large, it is convenient to think of the hit function *h*(*s*), defined over *s* ∈ (0,1), as a continuous function. We further assume that it is differentiable almost everywhere. This allows the use of calculus to discuss various concepts. The collection of points {*s, h(s*)}, traces out a so-called hit curve. For simplicity, the hit function *h*(*s*) is also referred to as the hit curve. Similar to the ROC curve, the hit curve is a signature of the underlying test’s effectiveness.

The AP is defined as the PPV averaged over the true positive fractions (TPFs). Using the notations defined above,

$$\begin{array}{llll} & \mathrm{PPV}\left(s\right)=\frac{h\left(s\right)}{s}=\frac{m\left(k\right)}{d\left(k\right)},\; & & \;\\ & \mathrm{TPF}\left(s\right)=\frac{h\left(s\right)}{\pi }=\frac{m\left(k\right)}{N_{1}},\; & & \; \end{array}$$

and

(1) $$\mathrm{AP}\equiv \int_{0}^{1}\mathrm{PPV}\left(s\right)\mathrm{dTPF}\left(s\right)=\frac{1}{\pi }\int_{0}^{1}\frac{h\left(s\right)}{s}\mathrm{dh}\left(s\right).$$

The ROC curve refers to the collection of points {FPF(s), TPF(s)}, where “FPF” stands for the false positive fraction – in particular,

$$\mathrm{FPF(}s)=\frac{s-h\left(s\right)}{1-\pi }=\frac{d\left(k\right)-m\left(k\right)}{N-N_{1}}.$$

Thus, the AUC is given by

(2) $$\mathrm{AUC}\equiv \int_{0}^{1}\mathrm{TPF}\left(s\right)\mathrm{dFPF}\left(s\right)=\frac{1}{\pi (1-\pi )}\left[\int_{0}^{1}h(s)\text{ds}-\frac{\pi^{2}}{2}\right].$$

The derivations for the last equality in both Eqs 1 and 2 are given in Supplementary Material.

For those not familiar with either of these concepts, they are often abstract at first sight and a few examples are warranted. For those already comfortable with the ideas, the next two subsections can be skipped.

#### A random test

If a test is random, then *h*(*s*)* =* π*s*. That is, the true positive rate stays constant at π, the overall proportion of diseased subjects. By Eqs 1 and 2, we have

$$\begin{array}{llll} \mathrm{AP(Random)} & =\frac{1}{\pi }\int_{0}^{1}\frac{\pi s}{s}d\pi \text{s}=\pi ,\; & & \;\\ \mathrm{AUC(Random)} & =\frac{1}{\pi (1-\pi )}\left[\int_{0}^{1}\pi \text{sds}-\frac{\pi^{2}}{2}\right]=\frac{1}{2}. \end{array}$$

#### A perfect test

If a test is perfect, then

h(s)=s,whens≤π;π,when s>π.

That is, the positive predictive rate is 100% until all diseased subjects have been identified, after which the positive predictive rate necessarily stays at zero.

By Eqs 1 and 2, we have

$$\begin{array}{llll} \mathrm{AP(Perfect)} & =\frac{1}{\pi }\int_{0}^{\pi }\frac{s}{s}\mathrm{ds}+\int_{\pi }^{1}\frac{\pi }{s}\times 0\times \mathrm{ds = 1},\; & & \;\\ \mathrm{AUC(Perfect)} & =\frac{1}{\pi \text{ (1– }\pi \text{)}}\left[\int_{0}^{\pi }\mathrm{sds+}\int_{\pi }^{1}\pi \text{ds}-\frac{\pi^{2}}{2}\right]=1. \end{array}$$

### Sample version and discrete scores

Conceptually, it is convenient to think of *h*(*s*) as a smooth continuous curve, and it makes sense for a hypothetical population where *N* can be infinitely large and the test score is of a continuous nature. In practice, however, we are often dealing with data obtained from a sample that gives discrete test scores, or data from an ordinal scored test, giving rise to a “ragged” hit curve. In this section, we describe the discrete set-up and derive explicit expressions for the AUC and the AP under this set-up.

Suppose that a screening test gives *K* distinct scores for a sample of *n* subjects. When *K* < *n*, it means that some subjects’ scores are tied. The case of “no ties” corresponds to the special case of *K =* *n*. With *K* distinct scores, the subjects are partitioned into *K* groups. Within each group, some are diseased and the others are non-diseased, but they cannot be distinguished by the test score. We use *r*_1_ to denote the set of all subjects receiving the top score, *r*_2_ to denote the set of all subjects receiving the next top score, and so on for *r*_3_, …, *r*_K_. Furthermore, let

*S_k_* = total number of subjects in *r_k_*,
*Z_k_* = total number of diseased subjects in *r_k_*,
${\bar{Z}}_{k}=S_{k}-Z_{k}$, total number of non-diseased subjects in *r_k_*.

Table 2 summarizes the set-up and these notations. Under the typical set-up (Table 2), if we threshold the scores at *x*_k_, then all those in partitions *r*_1_, *r*_2_, …, *r*_k_ will be declared diseased, and the rest declared non-diseased. Therefore, we have

$$d(k)=\sum_{k^{\prime }\leq \text{k}}S_{k\prime }\;\mathrm{and}\;m(k\mathrm{) =}\sum_{k^{\prime }\leq \text{k}}Z_{k^{\prime }}$$

**Table 2:** **A screening test partitions a sample of *n* subjects into K groups (K distinct scores)**.

Score	*x*_1_ > *x*_2_ > … > *x*_k_ > *x*_k+1_ > … > *x*_K_	Total
Partition	*r*_1_ *r*_2_ … *r*_k_ ¦ *r*_k+1_ … *r*_k_	
Diseased	*Z*_1_ *Z*_2_ … *Z*_k_ ¦ *Z*_k+1_ … *Z*_k_	*n*_1_
Non-diseased	${\bar{Z}}_{1}\;{\bar{Z}}_{2}\;\cdots \;{\bar{Z}}_{\text{k}}\;\;¦\;\;\;{\bar{Z}}_{\text{k}+1}\;\cdots \;{\bar{Z}}_{\text{k}}$	*N*_0_
Total	*S*_1_ *S*_2_ … *S*_k_ ¦ *S*_k+1_ … *S*_k_	*n*

*The broken bars (¦) illustrates the case where all those with scores ≥*x*_k_ (left) are predicted to be diseased, while all those with scores <*x*_k_ (right) are predicted to be non-diseased*.

As a result, the AP, as expressed by Eq. 1 as an integral, can be approximated with a summation, and the summands can be further rearranged, so that overall the AP is expressed as a weighted density function of the diseased subjects, where the weights are the positive predicted values, PPV(*k*), denoted by *w_k_* in the following equation:

(3) $$\begin{array}{llll} \hat{\mathrm{AP}} & =\frac{1}{n_{1}∕n}\sum_{k=1}^{K}\frac{m\left(k\right)}{d\left(k\right)}\cdot \frac{\Delta m\left(k\right)}{n}\; & & \;\\ & =\frac{1}{n_{1}}\sum_{k=1}^{K}\frac{m\left(k\right)}{d\left(k\right)}Z_{k}\; & & \;\\ & =\underset{w_{1}}{\underset{︸}{\left[\frac{Z_{1}}{S_{1}}\right]}}\left[\frac{Z_{1}}{n_{1}}\right]+\underset{w_{2}}{\underset{︸}{\left[\frac{Z_{1}+Z_{2}}{S_{1}+S_{2}}\right]}}\left[\frac{Z_{2}}{n_{1}}\right]+\cdots +\underset{w_{K}}{\underset{︸}{\left[\frac{Z_{1}+Z_{2}+\cdots +Z_{K}}{S_{1}+S_{2}+\cdots +S_{K}}\right]}}\left[\frac{Z_{K}}{n_{1}}\right]\; & & \;\\ & =\sum_{k=1}w_{k}\left[\frac{Z_{k}}{n_{1}}\right]. \end{array}$$

Likewise, the AUC, as expressed by Eq. 2 as an integral, can also be approximated as a summation, and its summands can also be further rearranged, so that overall the AUC is also expressed as a weighted density function of the diseased subjects, a form similar to the final expression of Eq. 3:

$$\begin{array}{llll} \hat{\mathrm{AUC}} & =\frac{1}{\left(n_{1}∕n\right)\left(n_{0}∕n\right)}\left\{\sum_{\text{k}=\text{1}}^{\text{K}}\left[\frac{m\left(k\right)}{n}\right]\left[\frac{\Delta d\left(k\right)}{n}\right]-\frac{1}{2}{\left(\frac{n_{1}}{n}\right)}^{2}\right\}\; & & \;\\ & =\frac{n}{n_{0}}\left\{\sum_{\text{k}=\text{1}}^{\text{K}}\left[\frac{m\left(k\right)}{n_{1}}\right]\left[\frac{\Delta d\left(k\right)}{n}\right]\right\}-\frac{1}{2}\frac{n_{1}}{n_{0}},\; & & \; \end{array}$$

where the term inside the curly brackets is

(4) $$\begin{array}{llll} & \sum_{\text{k}=\text{1}}^{\text{K}}\left[\frac{m\left(k\right)}{n_{1}}\right]\left[\frac{\Delta d\left(k\right)}{n}\right]\; & & \;\\ & \;=\left[\frac{Z_{1}}{n_{1}}\right]\left[\frac{S_{1}}{n}\right]+\left[\frac{Z_{1}+Z_{2}}{n_{1}}\right]\left[\frac{S_{2}}{n}\right]+\cdots +\left[\frac{Z_{1}+Z_{2}+\cdots +Z_{K}}{n_{1}}\right]\left[\frac{S_{K}}{n}\right]\; & & \;\\ & \;=\underset{w_{1}^{\prime }}{\underset{︸}{\left[\frac{S_{1}+S_{2}+\cdots +S_{K}}{n}\right]}}\left[\frac{Z_{1}}{n_{1}}\right]+\underset{w_{2}^{\prime }}{\underset{︸}{\left[\frac{S_{2}+\cdots +S_{K}}{n}\right]}}\left[\frac{Z_{2}}{n_{1}}\right]+\cdots +\underset{w_{k}^{\prime }}{\underset{︸}{\left[\frac{S_{K}}{n}\right]}}\left[\frac{Z_{K}}{n_{1}}\right]\; & & \;\\ & \;=\sum_{\text{k}=1}^{\text{K}}w_{k}^{\prime }\left[\frac{Z_{k}}{n_{1}}\right]. \end{array}$$

Equations 3 and 4 give convenient and explicit expressions for estimating the AP (and the AUC) in practice. They also reveal that both the AP and AUC can be expressed as weighted averages of *Z*_1_, *Z*_2_, …, *Z*_K_, except that they use different weights: *w*_k_ for the AP and *w’*_k_ for the AUC.

## Connections between AP and AUC

The expressions in Eqs 3 and 4 indicate that the AP places more emphasis on initial true positives than does the AUC. To see this, let us look at the weights *w*_k_ and *w’*_k_, which differentiate the two measures. The difference between these two weights can be seen most clearly in the case of “no ties,” i.e., *K* = *n*. Under such circumstances, each *r_k_* contains just one subject, so *S_k_ =* 1 for all *k*, and each *Z*_k_ is either zero or one.

Then, from Eq. 3, *w_k_* for the AP is given by

$$\left[\sum {\;}_{\text{i}=\text{1}}^{\text{k}}\;Z_{\text{i}}\right]∕\left[\sum {\;}_{\text{i}=\text{1}}^{\text{k}}\;S_{\text{i}}\right],$$

where *Z*_i_ = 1 or 0 and *S*_i_ = 1 for *i* = 1, …, *k*. So

$$\sum_{\text{i}=1}^{\text{k}}Z_{\text{i}}\text{ = number of true positive up to}\;k=m\left(k\right)$$

and $\sum_{i=1}^{k}S_{\text{i}}=k$. Thus

(5) $$\begin{array}{ll} w_{k} & =\frac{\sum_{1}^{k}Z_{i}}{\sum_{1}^{k}S_{i}}=\frac{m\left(k\right)}{k}=\frac{\text{number of true positives up to }k}{k}\equiv \mathrm{PPV}\left(k\right). \end{array}$$

Similarly, from Eq. 4, $w_{\text{k}}^{\prime }$ for the AUC is given by $\sum_{\mathrm{i=k}}^{k}S_{i}∕n$. Since *S*_i_ = 1 for *i* = *k*, *k* + 1, …, *n*, we have $\sum_{\mathrm{i=k}}^{k}S_{i}=n-\left(k-1\right)$, and

(6) $$w_{k}^{\prime }=\frac{\sum_{\mathrm{i=k}}^{k}S_{i}}{n}=\frac{n-\left(k-1\right)}{n}.$$

It is clear from Eqs 5 and 6 that the weights used by AUC (*w’*_k_) are independent of, whereas the ones used by the AP (*w*_k_) are adaptive to, the predictive performance of the test itself.

Suppose that we are comparing two tests, A and B, and a diseased subject is ranked at *k* (i.e., *Z*_k_ = 1) by both tests. When estimating the AUC for the two different tests, the diseased subject will receive a fixed weight (*n* − *k* + 1)/*n*, in both tests A and B. When estimating the AP, however, the weight the subject receives will depend on the strength of the test itself. In particular, if test A identified more diseased subjects before *k* than did test B, the relative weight on *Z_k_* would be bigger for test A than for test B. This shows that the AP places more emphasis on early true positives than does the AUC.

## Asymptotic Variance

To use the AP as a performance metric in practice, we derived an asymptotic variance formula for the estimated AP so that inferences can be made. Supplementary Material contains the detailed derivations. Here, we illustrate the finite sample property of our asymptotic variance formula using data from the Digital Mammographic Imaging Screening Trial (14), which compared digital versus film mammography for breast cancer screening.

Over 42,000 women were enrolled in the trial and underwent both digital and film mammography. Using a seven-point malignancy scale, each pair of mammograms was rated separately by two independent radiologists. At 15-month follow-up, a total of 335 breast cancers were confirmed in the final cohort, and the question was: which type of mammography is better at detecting these cases of cancer?

We analyzed the data reported in Table 3 by Pisano et al. (14), which is shown below. The estimated AUC and AP for the two technologies are given in Table 4, together with several SE estimates of the AP. Here, we can see that the asymptotic estimates of SEs do agree closely with standard bootstrap estimates (15), indicating that our variance formula performs well on finite samples.

**Table 3:** **Diagnostic accuracy of digital and film mammography using a seven-point malignancy scale after 455 days of follow-up [adapted from Table 3 of Pisano et al.(14)]**.

Malignancy score		7	6	5	4	3	2	1	Total
Digital	Category total	11	29	69	1061	2224	6588	32588	42570
	Cancers	10	18	25	85	49	25	122	334
Film	Category total	17	29	70	942	2291	6910	32486	42745
	Cancers	13	24	25	74	35	33	131	335

**Table 4:** **Breast cancer example (see Asymptotic Variance)**.

Mammography type	AUC	AP	SE of AP		
			Asymptotic	P-bootstrap	NP- bootstrap
Digital	0.753	0.144	0.0197	0.0197	0.0194
Film	0.735	0.166	0.0219	0.0216	0.0215

*Film versus digital mammography. P-bootstrap, parametric bootstrap; NP-bootstrap, non-parametric bootstrap. A total of 5000 bootstrap samples were generated for each bootstrap method*.

Overall, digital mammography fared slightly better than film mammography on the AUC-scale, but the AP favored film mammography slightly over digital mammography. The difference in the AP (or the AUC) between the two types of mammography was relatively small and there is likely no clinically significant difference between the two tests.

## Discussion

In this paper, we derived explicit expressions for and examined the connections between the AUC and the AP (see Definitions and Connections Between AP and AUC). We compared these metrics in two screening settings: the prostate cancer biomarker (Figure 2) is an example of a test with continuous scores; and the breast cancer mammogram (Table 3) is an example of a test with ordinal scores. We also derived an asymptotic variance formula for the estimated AP.

Our objective is to show that the AP has advantages over the AUC when evaluating screening tests as opposed to diagnostic tests at the pre-clinical stage, when possibly many different candidate tests (or biomarkers) are considered. It is well known that the AUC measures the discriminative ability (the separation of two probability density functions) of the test scores for diseased and non-diseased subjects, and that the AUC has a conditional probability interpretation – given a randomly selected pair of diseased and non-disease subjects, the AUC is the probability that the test assigns a higher risk score to the diseased subject. However, we can think of five issues important to evaluating a screening test that are not properly addressed by the AUC metric:

(1) When prevalence is low, the false positive rate needs to be low for a useful screening test to be acceptable (10). A larger AUC does not guarantee this as shown by the prostate cancer biomarker example (see Illustrative Example).
(2) If we randomly sampled two individuals from the population when prevalence is low, it is unlikely that we would obtain a pair consisting of a diseased individual and a non-diseased one. Therefore, the conditional probability interpretation of the AUC is not directly relevant to the screening task *per se*.
(3) Hypothetically, for two respective populations with high and low prevalence rates of the same disease, the best screening test to use in each case could be different. However, the AUC will choose one single test for both populations regardless of the prevalence, which may not be the best screening test for either population.
(4) While the PPV of a test is of considerable clinical interest, the AUC does not contain information about the PPV.
(5) For patients, the ability of the screening test to predict their disease status (i.e., the PPV) is an idea easier to understand and relate to than the idea of diagnostic accuracy. The predicted risk facilitates shared medical decision making (16), which is a core concept for patient-centered care.

The AP, on the other hand, takes into account not only the separation of the two density curves but also how they separate, and the *prevalence* of the specific disease. It directly addresses the aforementioned issues (3–5), and indirectly addresses the issue (1). The last point can be vividly illustrated with an example from Wald and Bestwick (10).

By fixing the test scores of non-diseased individuals to be normally distributed with mean 0 and SD 1, and varying the mean and SD for the diseased individuals, Wald and Bestwick (10) were able to construct tests having the same AUC but vastly different detection rates (DRs) at given false positive levels, and vice versa. We took the example given in their Figure 2 and estimated the AP using the same three prevalence rates, 0.5, 0.09, and 0.01, as we did in Table 1. The results are shown in Table 5. First, we can see that the AP distinguishes the performance of the three tests and ranks the three tests in the same order as the DR and FPF do. Moreover, on the AP-scale, the advantage of the best test becomes more prominent as the prevalence rate decreases. The DR and FPF, however, remain the same and so does the AUC, because they are independent of the prevalence rate.

**Table 5:** **AUC, AP, DR, and FPF for three tests from Wald and Bestwick [(10), Figure 2]**.

	AUC^a^	AP			DR at FPF 0.05^a^	FPF at DR 50%^a^
		π = 0.5	π ≈ 0.09	π ≈ 0.01	
SD_A_ = SD_U_	0.75	0.74	0.26	0.04	0.24	0.17
SD_A_ = 1.5SD_U_	0.75	0.79	0.42	0.16	0.39	0.11
SD_A_ = 2SD_U_	0.75	0.81	0.51	0.29	0.47	0.07

*^a^Numbers are from Tables 1–3 of Wald and Bestwick (10) for AUC = 0.75, a setting illustrated further in their Figure 2. DR, detection rate, the proportion of diseased individual identified at a given threshold. FPF, False positive fraction, the proportion of non-diseased individual identified at a given threshold. The APs are averages over 1000 simulations, using *n* = 10000 for non-diseased subjects, and *n* = 100 (π ≈ 0.01), 1000 (π ≈ 0.09), and 10000 (π = 0.5) for diseased subjects, respectively. SD_A_ = SD of affected (diseased) individuals; SD_U_ = SD of unaffected (non-diseased) individuals*.

As a weighted average of PPV, the AP measures the overall positive predictive power of a screening test, which is used to predict disease status for individual patients in a target population with a specific prevalence rate. Typically, the disease prevalence rate is low for a screening test, and we naturally would like to avoid raising too many red flags, but for the precious few flags that we do raise (i.e., the top-ranked subjects), we would like to detect as many true positives as possible. In our opinion, the AP is better aligned than the AUC is with the goal of assessing the positive predictive ability of a screening test^1^.

Taking breast cancer screening and diagnostic tests as an example, the screening and diagnostic mammography are exactly the same technology; the only difference is their respective target populations – without or with suspicion of breast cancer. In the clinical trial example (see Asymptotic Variance), the disease was diagnosed in 0.78% of the screening population at 15 months post-screening (0.59% at 12 month post-screening). Radiologists do explicitly consider the very low prevalence rate when assigning malignancy scores to avoid too many false positives (17). In other words, the predictive value of a screening test is of clinical interest, and clinicians may very well prefer a screening test that is favored by the AP to one favored by the AUC.

Consider again the prostate cancer biomarker example (see Illustrative Example). For pair B (9149.121, 5074.164), when the “prevalence rate” was artificially set at 50% (by virtue of the case–control design), the marker 5074.164 scored higher on the AUC-scale; but on the AP-scale the two biomarkers were similar. However, when the prevalence was reduced to better mimic the screening setting in real life, the AP started to favor the other marker, 9149.121, by a substantial margin, thus supporting the use of marker 9149.121 as a screening tool over marker 5074.164. For assessing screening (as opposed to diagnostic) tests, therefore, a performance metric that emphasizes the test’s overall predictive ability for individual patients in the targeted screening population, such as the AP, could improve decision making.

One may argue that the partial AUC addresses the very concern that not all parts of the ROC curve are relevant, so a new metric such as the AP is not needed. We think that, in order to use the partial AUC, a subjective threshold is still needed that typically incorporates additional information such as the prevalence and relative costs of false positives and false negatives. These relative costs are hard to assess in practice, and often arbitrary and subjective. In addition, with the partial AUC, the appealing probability interpretation of the AUC is also lost. We have observed that, in clinical research, the partial AUC has not been used as often as it should have been. To this effect, we think that the threshold-free AP metric offers an attractive alternative to the partial AUC.

Finally, we think that the AP is useful not only for medical screening tests but also for the risk prediction of low probability events in general. Often, models are constructed and covariates are selected in order to predict some future event in a specific population, e.g., the risk of having a cardiovascular event in the next 10 years, or the risk of having a secondary neoplasm in the next 10 years for cancer survivors. One of the main objectives is to identify patients who have a high risk of developing these conditions. Since many of these events have low probabilities, the AP may be a useful performance measure for reasons similar to those discussed above. Currently, however, prediction models and competing risk factors are almost exclusively assessed by ROC curves and more specifically, by the AUC (18).

## Conflict of Interest Statement

The authors declare that the research was conducted in the absence of any commercial or financial relationships that could be construed as a potential conflict of interest.

## Supplementary Material

The Supplementary Material for this article can be found online at http://journal.frontiersin.org/article/10.3389/fpubh.2015.00057/abstract
