# Supplementary material for: Threshold-Free Measures for Assessing the Performance of Medical Screening Tests
Source: Front Public Health. 2015 Apr 20;3:57. doi: 10.3389/fpubh.2015.00057 (PMC4403252; doi:10.3389/fpubh.2015.00057)
Supplement: Supplementary file 1 [file Datasheet_1.PDF]

Appendix A: Derivation of Eqs. (1) and (2)

Recall that

$\pi \equiv N_1 / N$ = P(Y =1),

$s \equiv$ d(k) / N = P(X ≥ x_k), and

h(s) ≡ m(k) / N = P(Y=1, X ≥ x_k),

where d(k) in the number of subjects with scores greater than or equal to x_k, and m(k) is the number of truly diseased subjects in those d(k) subjects.

By definition, the positive predicted value (PPV) and the true positive fraction (TPF) at s can be expressed as

$$PPV(s) = \frac{h(s)}{\mathrm{s}} = \frac{m(k)}{d(k)},$$

$$TPF(s) = \frac{h(s)}{\pi} = \frac{m(k)}{N_1}.$$

Again, by the definition of the AP,

$$AP \equiv \int_0^1 PPV(s) dTPF(s)$$

$$= \int_0^1 \frac{h(s)}{s} \times \frac{dh(s)}{\pi}$$

$$= \frac{1}{\pi} \int_0^1 \frac{h(s)}{s} dh(s)$$

To derive the formula for the AUC, we first express the false positive fraction (FPF) at s as

$$FPF(s) = \frac{s - h(s)}{1 - \mathrm{\pi}} = \frac{d(k) - m(k)}{N - N_1}.$$

Thus

$$AUC \equiv \int_0^1 TPF(s) dFPF(s)$$

$$= \int_0^1 \frac{h(s)}{\mathrm{\pi}} d\left[\frac{s - h(s)}{1 - \pi}\right]$$

$$= \frac{1}{\pi(1 - \pi)} \int_0^1 h(s)[ds - dh(s)]$$

$$= \frac{1}{\pi(1 - \pi)} \left[\int_0^1 h(s) ds - \int_0^1 h(s) dh(s)\right]$$

$$= \frac{1}{\pi(1 - \pi)} \left[\int_0^1 h(s) ds - \frac{\pi^2}{2}\right]$$

The last equation is obtained using integration by parts, and the facts that h(1) = P(Y=1) = π and h(0)=0.

Appendix B: Derivation of Asymptotic Variance Formula

Notice that the data in Table 2 follow the ensuing distributions:

$$(Z_1, Z_2, \cdots, Z_K)|n_1 \sim \text{multinomial}(n_1; p_1, p_2, \cdots, p_K),$$
$$(\overline{Z}_1, \overline{Z}_2, \cdots, \overline{Z}_K)|n_1 \sim \text{multinomial}(n - n_1; q_1, q_2, \cdots, q_K),$$
$$n_1 \sim \text{binomial}(n, \pi), \qquad (7)$$

where

$$p_k = \int_{r_k} f_1(x)\, dx, \;\; q_k = \int_{r_k} f_0(x)\, dx,$$

and $f_1(x)$, $f_0(x)$ are respectively the probability density distributions of the scores for the diseased and non-diseased subjects. In addition,

$$(Z_1, Z_2, \cdots, Z_K) \perp (\overline{Z}_1, \overline{Z}_2, \cdots, \overline{Z}_K)|n_1.$$

The log-likelihood function (aside from a constant) is given by

$$\ell(\mathbf{p}, \mathbf{q}, \pi) = \sum_{k=1}^{K} z_k \log p_k + \sum_{k=1}^{K} \overline{z}_k \log q_k + [n_1 \log \pi + (n - n_1) \log(1 - \pi)], \quad (8)$$

where $\boldsymbol{p} \equiv (p_1, \cdots, p_K)^{\mathrm{T}}, \boldsymbol{q} \equiv (q_1, \cdots, q_K)^{\mathrm{T}}$,

$$\sum_{k=1}^{K} p_k = 1, \quad \sum_{k=1}^{K} q_k = 1, \quad \sum_{k=1}^{K} z_k = n_1, \quad \text{and} \sum_{k=1}^{K} \overline{z}_k = n - n_1.$$

Let $(\hat{\boldsymbol{p}}, \hat{\boldsymbol{q}}, \hat{\pi})$ denote the maximum likelihood estimates (MLEs) of $(\boldsymbol{p}, \boldsymbol{q}, \pi)$. Then, by classical theory of the MLEs,[19] the asymptotic variance of $(\hat{\boldsymbol{p}}, \hat{\boldsymbol{q}}, \hat{\pi})$ is simply $\mathbf{J}^{-1}$, where **J** is the Fisher information matrix associated with the log-likelihood function (8). Since the AP, as expressed by

Eq. (4), can be written as a function of $(\widehat{\boldsymbol{p}}, \widehat{\boldsymbol{q}}, \hat{\pi})$, i.e., $\widehat{AP} = g(\widehat{\boldsymbol{p}}, \widehat{\boldsymbol{q}}, \hat{\pi})$, we can simply apply the delta method to estimate its asymptotic variance,

$$\widehat{\mathrm{Var}}\left(\widehat{\mathrm{AP}}\right) = (\nabla g)^{\mathrm{T}} \hat{\mathbf{J}}^{-1} (\nabla g), \qquad (9)$$

where $\hat{\boldsymbol{J}}$ denotes the *observed* Fisher information matrix.

More specifically, since

$$\hat{p}_k = \frac{Z_k}{n_1}, \hat{q}_k = \frac{\overline{Z}_k}{n - n_1}, \hat{\pi} = \frac{n_1}{n},$$

we can write

$$\begin{aligned} \widehat{AP} &= g(\widehat{\boldsymbol{p}}, \widehat{\boldsymbol{q}}, \hat{\pi}) \\ &= \sum_{k=1}^{K} \left[\hat{p}_k \left(\frac{\hat{\pi} \sum_{k' \le k} \hat{p}_{k'}}{\hat{\pi} \sum_{k' \le k} \hat{p}_{k'} + (1 - \hat{\pi}) \sum_{k' \le k} \hat{q}_{k'}} \right) \right]. \end{aligned}$$

Let $\theta \equiv (\boldsymbol{p}, \boldsymbol{q}, \pi)$ and $\hat{\theta} = (\widehat{\boldsymbol{p}}, \widehat{\boldsymbol{q}}, \hat{\pi})$. Due to the multinomial constraints, we have $p_1 + p_2 + \cdots p_K = 1$ and $q_1 + q_2 + \cdots q_K = 1$. In practice, we work with (K-1)-dimensional vectors, $\boldsymbol{p}$ and $\boldsymbol{q}$. By direct algerbraic calculations, we can obtain that

$\hat{\mathrm{J}} = \mathrm{E}\left[-\frac{\partial l^2}{\partial \theta \partial \theta^{\mathrm{T}}}\right] = \begin{bmatrix} P & - & - \\ - & Q & - \\ - & - & a \end{bmatrix}$, where P, Q are (K-1)×(K-1) matrices with

$$P_{kk} = \frac{z_k}{\hat{p}_k^{\ 2}} + \frac{z_K}{\hat{p}_K^{\ 2}} \quad \text{for } k = 1,2, \cdots K - 1$$

$$P_{kk'} = \frac{z_K}{\hat{p}_K^{\ 2}} \qquad \text{for all } k \neq k'$$

$$Q_{kk} = \frac{\overline{z}_k}{\hat{q}_k^{\ 2}} + \frac{\overline{z}_K}{\hat{q}_K^{\ 2}} \quad \text{for } k = 1,2, \cdots K - 1$$

$$Q_{kk'} = \frac{\overline{z}_K}{\hat{q}_K{}^2} \qquad \text{for all } k \neq k'$$

and

$$a = \frac{n_1}{\hat{\pi}^2} + \frac{n - n_1}{(1 - \hat{\pi})^2}.$$

Now let

$$P_k = \sum_{k' \leq k} \hat{p}_{k'} \quad Q_k = \sum_{k' \leq k} \hat{q}_{k'}$$

and

$$C_k = \hat{\pi} P_k + (1 - \hat{\pi}) Q_k.$$

Again, by algebraic calculations, we can obtain that

$$\nabla g = \begin{bmatrix} \nabla_{\boldsymbol{p}} \\ \nabla_{\boldsymbol{q}} \\ \nabla_{\pi} \end{bmatrix},$$

where $\nabla_{\boldsymbol{p}}, \nabla_{\boldsymbol{q}}$ are (K-1) dimensional vectors with

$$\nabla_{\boldsymbol{p}}(k) \equiv \frac{\partial g}{\partial \hat{p}_k} = \frac{\hat{\pi} P_k}{C_k} + \sum_{k'=k}^{K-1} \hat{p}_{k'} \left[\frac{\hat{\pi}(1 - \hat{\pi}) Q_{k'}}{C_{k'}{}^2} \right] - \hat{\pi},$$

$$\nabla_{\boldsymbol{q}}(k) \equiv \frac{\partial g}{\partial \hat{q}_k} = \sum_{k'=k}^{K-1} \hat{p}_{k'} \left[\frac{-\hat{\pi}(1 - \hat{\pi}) P_{k'}}{C_{k'}{}^2} \right] \text{ for } k = 1, \cdots, K - 1,$$

and

$$\nabla_{\pi} = \frac{\partial g}{\partial \hat{\pi}} \sum_{k=1}^{K} \hat{p}_k \left[\frac{P_k Q_k}{C_k{}^2} \right].$$

Putting all pieces together, the asymptotic variance can be computed as

$$\widehat{\mathrm{Var}}\left(\widehat{\mathrm{AP}}\right) = (\nabla g)^{\mathrm{T}} \hat{\mathbf{j}}^{-1} (\nabla g).$$
